# Continental-scale animal tracking reveals functional movement classes across marine taxa

**DOI:** 10.1038/s41598-018-21988-5

**Published:** 2018-02-27

**Authors:** Stephanie Brodie, Elodie J. I. Lédée, Michelle R. Heupel, Russell C. Babcock, Hamish A. Campbell, Daniel C. Gledhill, Xavier Hoenner, Charlie Huveneers, Fabrice R. A. Jaine, Colin A. Simpfendorfer, Matthew D. Taylor, Vinay Udyawer, Robert G. Harcourt

**Affiliations:** 1 School of Biological, Earth and Environmental Sciences, University of New South Wales, Sydney, NSW 2052 Australia; 2 Sydney Institute of Marine Science, Chowder Bay Road, Mosman, NSW 2088 Australia; 3 Institute of Marine Science, University of California Santa Cruz, Santa Cruz, CA 95064 USA; 4 Centre for Sustainable Tropical Fisheries and Aquaculture & College of Marine and Environmental Sciences, James Cook University, Townsville, QLD 4811 Australia; 5 Australian Institute of Marine Science, Townsville, QLD 4810 Australia; 6 CSIRO Oceans and Atmosphere, Dutton Park, QLD 4102 Australia; 7 School of Environment, Charles Darwin University, Casuarina, NT 0909 Australia; 8 CSIRO Oceans and Atmosphere and CSIRO National Research Collections Australia, Hobart, TAS 7000 Australia; 9 Australian Ocean Data Network, Integrated Marine Observing System, University of Tasmania, Private Bag 110, Hobart, TAS 7001 Australia; 10 School of Biological Sciences, Flinders University, Adelaide, SA 5042 Australia; 11 Department of Biological Sciences, Macquarie University, Sydney, NSW 2109 Australia; 12 Port Stephens Fisheries Institute, New South Wales Department of Primary Industries, Taylors Beach, NSW 2316 Australia; 13 Arafura Timor Research Facility, Australian Institute of Marine Science, Darwin, NT 0810 Australia

## Abstract

Acoustic telemetry is a principle tool for observing aquatic animals, but coverage over large spatial scales remains a challenge. To resolve this, Australia has implemented the Integrated Marine Observing System’s Animal Tracking Facility which comprises a continental-scale hydrophone array and coordinated data repository. This national acoustic network connects localized projects, enabling simultaneous monitoring of multiple species over scales ranging from 100 s of meters to 1000 s of kilometers. There is a need to evaluate the utility of this national network in monitoring animal movement ecology, and to identify the spatial scales that the network effectively operates over. Cluster analyses assessed movements and residency of 2181 individuals from 92 species, and identified four functional movement classes apparent only through aggregating data across the entire national network. These functional movement classes described movement metrics of individuals rather than species, and highlighted the plasticity of movement patterns across and within populations and species. Network analyses assessed the utility and redundancy of each component of the national network, revealing multiple spatial scales of connectivity influenced by the geographic positioning of acoustic receivers. We demonstrate the significance of this nationally coordinated network of receivers to better reveal intra-specific differences in movement profiles and discuss implications for effective management.

## Introduction

Animal telemetry has transformed our ability to remotely-monitor animals and provide critical insights into how they utilize their environment, such as revealing new and unexpected behavior relating to fine-scale habitat use, home range extent, inter-specific interactions, phenology, and migratory patterns^1^. Monitoring individual animals has revealed high intra-specific variability in behavior^2,3^, yet commonalities in movement patterns exist and can persist across taxa^4^. Continued monitoring of multiple species across biomes can improve our understanding of intra- and inter-specific similarities and differences in animal movement ecology^3,4^. Animal-borne acoustic transmitters have become a common tool for remotely observing aquatic species. Large arrays of underwater hydrophones, termed acoustic receivers, are now deployed along many coastal areas worldwide, providing movement and habitat use data for acoustically-tagged fish and other marine species^1^

For most continents, geopolitical issues arising from cross-jurisdictional movements of valued species and resulting conflicts in resource use can complicate movement monitoring^5,6^. Such conflicts can reduce data sharing^7^, reduce co-management of natural resources^8^, and create barriers to incorporating movement data into biologically relevant management decisions^9,10^. Australia is the only continent whose entire coastline is under the jurisdiction of a single nation. Accordingly, this region provides a unique opportunity to examine the utility of a large-scale collaborative system for monitoring marine vertebrate movement ecology.

**Figure 1:**
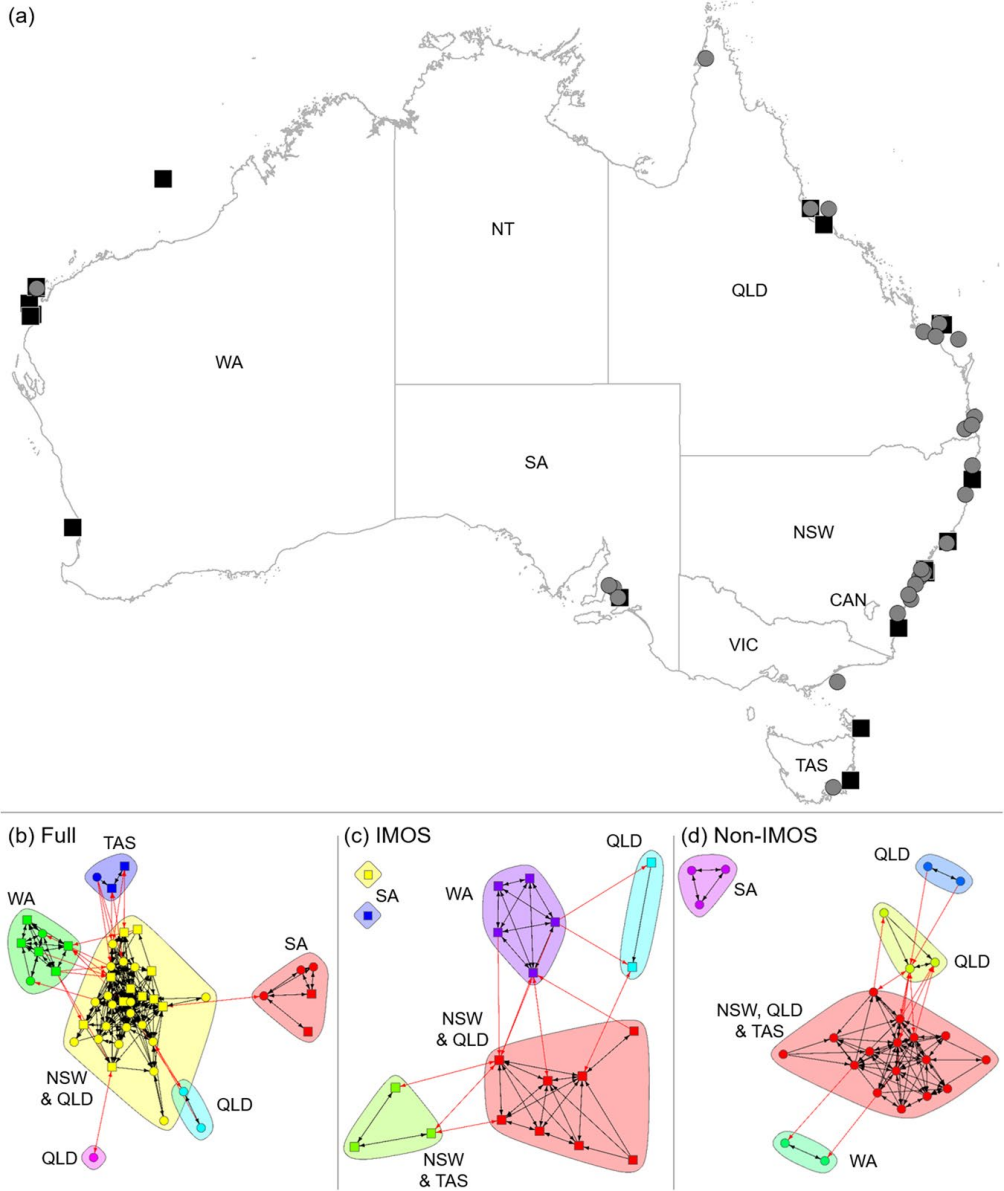
(**a**) Map of continental-scale acoustic telemetry installations around Australia with squares indicating IMOS installations and circles non-IMOS installations. Installations are considered to be a group of receivers deployed in a specific region. (**b**–**d**) Unique network clusters for each installation type. Clusters were a factor of geographic regions, indicated by state acronyms. Colors are not related between subplots. Arrows indicate connection across installations between (red) and within (black) clusters. IMOS installations in SA (**c**) have no connections and are considered isolate. The map of Australia was made in ArcMap 10.4.1, part of ArcGIS 10.4.1 for Desktop (http://desktop.arcgis.com/en/).

The Integrated Marine Observing System (IMOS), a multi-institutional collaboration funded by the Australian Government, has created a national ocean observing system that includes an animal telemetry platform for the Australian research community. The IMOS Animal Tracking Facility (IMOS ATF) facilitates large-scale, collaborative animal tracking research through the deployment of continental-scale curtains and grids of acoustic receivers^11^. This strategically located^11^, permanent array of acoustic receivers are integrated with a large number of independent, project-based, non-IMOS installations that are deployed by individual researchers and research teams to address regional research needs (Fig. 1a). An installation is considered to be a group of receivers deployed in a specific region. The integration of these installations in a network is achieved through a quality controlled, open-access repository for all associated data^12^. IMOS and independent research groups that contribute to IMOS all use acoustic telemetry equipment from Vemco (Nova Scotia, Canada), where all detections are from tags owned by independent research groups. The IMOS array was designed to inform management of long-ranging, cross-jurisdictional species that are exploited by fisheries or of conservation concern. Non-IMOS installations are regionally specific, but data is voluntarily integrated into the open-access repository to allow sharing of animal movement data across the continent. There is significant monetary and logistical support required to implement and maintain the IMOS ATF infrastructure and database repository. Thus, there is a need to evaluate the effectiveness of this collaborative national network in monitoring animal movement ecology, and to identify the spatial scales that the network operates over. Such an evaluation will demonstrate the utility of IMOS ATF to national user groups, and similar international acoustic telemetry platforms^13^.

We assessed the effectiveness of both the IMOS ATF and non-IMOS installations in monitoring animal movement using a ten-year data set collated from researchers, government institutions, universities, and IMOS. At the same time, we also examined the spatial scales the national network operates over, compared to regionally focused studies. Our approach used cluster analyses to classify movements based on when, where, and for how long animals were detected by receivers from IMOS ATF and non-IMOS installations. The analysis was based on transmitter detections only, with species de-identified so as to classify animal movement based on behavior rather than species groupings. The resultant classifications of marine animal movement are hereby termed functional movement class (FMC). Our approach to specifying FMCs highlight the plasticity of movement patterns across and within populations and species^4^, and have important implications for assessing species vulnerability to anthropogenic stressors. We also assessed the utility of, and redundancy within, the IMOS ATF by: a) undertaking network analyses on acoustic receiver installations, and b) comparing whether FMCs can be discerned when only IMOS or non-IMOS installations are included.

## Results

### Acoustic telemetry detection data

Approximately 35.8 million detections from 2181 individuals representing 92 species were extracted from the IMOS ATF data repository and analyzed. Of the 116 existing installations, 27 were IMOS and 89 were non-IMOS (i.e. independent research projects). Installations were not evenly distributed around Australia, with 87 installations on the east coast, 15 on the central part of the south coast, 1 on the north coast, and 13 installations on the west coast (Fig. 1a).

### Network Analysis of Installations

Network analyses assessed the utility and redundancy of the three installation types in the IMOS ATF: ‘IMOS’, ‘non-IMOS’, and ‘Full’ (includes both IMOS and non-IMOS). In the Full network analysis 48 installations were retained, with other installations excluded due to a lack of detections or movement between installations. The long-term data used in analyses ensures that valid inferences could be made on this partial network (i.e. 48 installations)^14^. Data from the Full installation formed a single network, with clusters based around geographic regions (Fig. 1b). Within the Full network, many of the IMOS installations had a high level of centrality and connectedness as indicated by high installation strength and eigenvalues (Table S1). Analysis of IMOS installations alone produced a simpler network (Fig. 1c; Table S2), while non-IMOS installations alone resulted in a network with fewer paths, greater average path length, lower density, and larger diameter (Fig. 1d; Table S2).

### Functional Movement Classes

**Figure 2:**
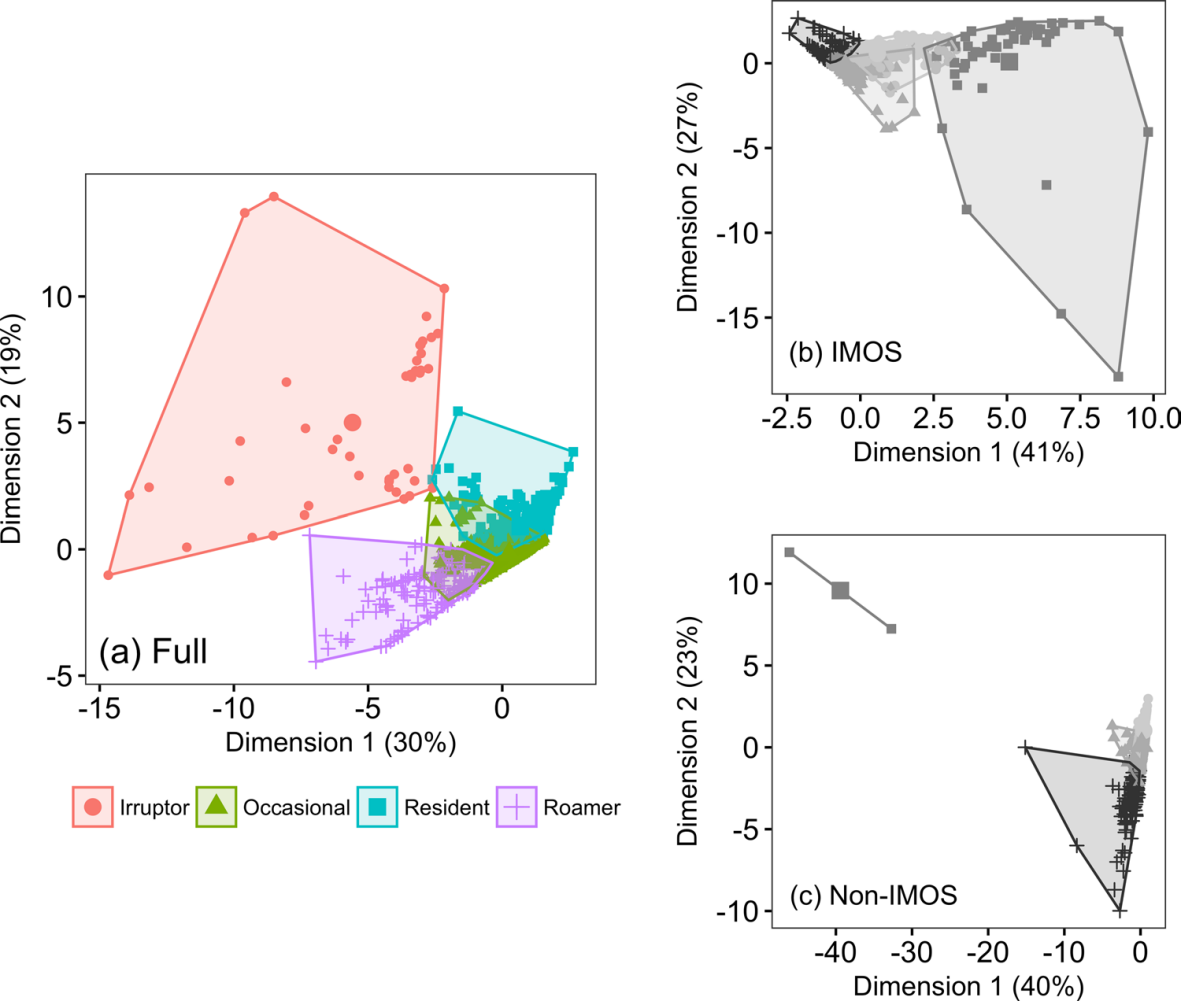
Functional movement classes (FMC) of acoustic telemetry detections, visualized using principle components. (**a**) Full installation detections with four clusters, as determined by a gap-statistic; (**b**) IMOS only detections required to have four clusters despite the gap statistic indicating one cluster; (**c**) Non-IMOS detections required to have four clusters despite the gap statistic indicating one cluster. Grey colors in (**b**) and (**c**) indicate that four FMCs cannot be discerned when individually examining IMOS and non-IMOS installations alone. Percentages on axes indicate the percent variance explained by that dimension.

**Figure 3:**
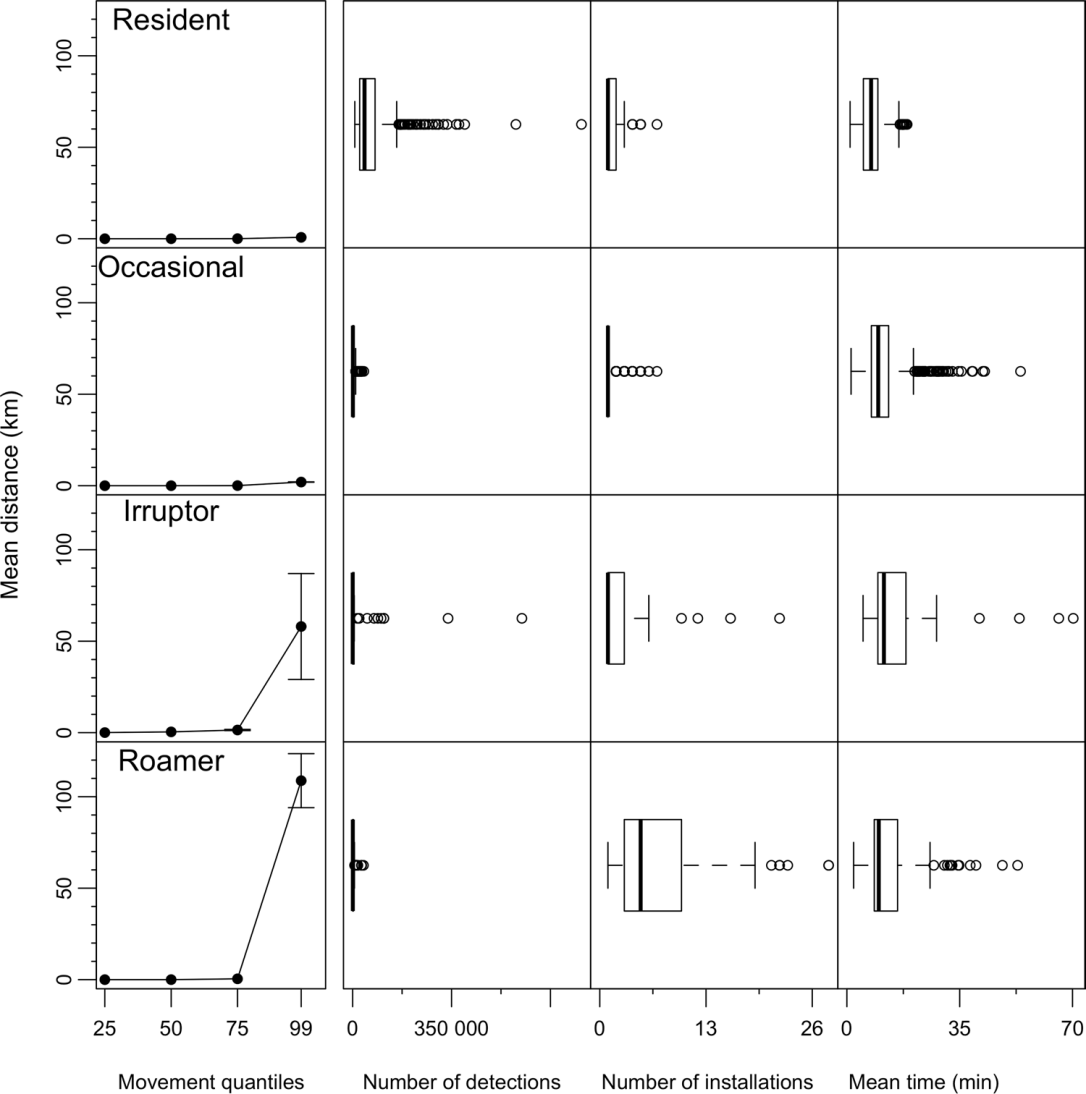
Distribution of covariates (columns) that describe each functional movement class (FMC; rows). First column is the mean distance (km) for the 25%, 50%, 75%, 99% movement quantiles. Remaining columns are boxplots indicating the distribution of covariates: number of detections, number of installations at which a transmitter was detected, and the mean time between detections.

**Figure 4:**
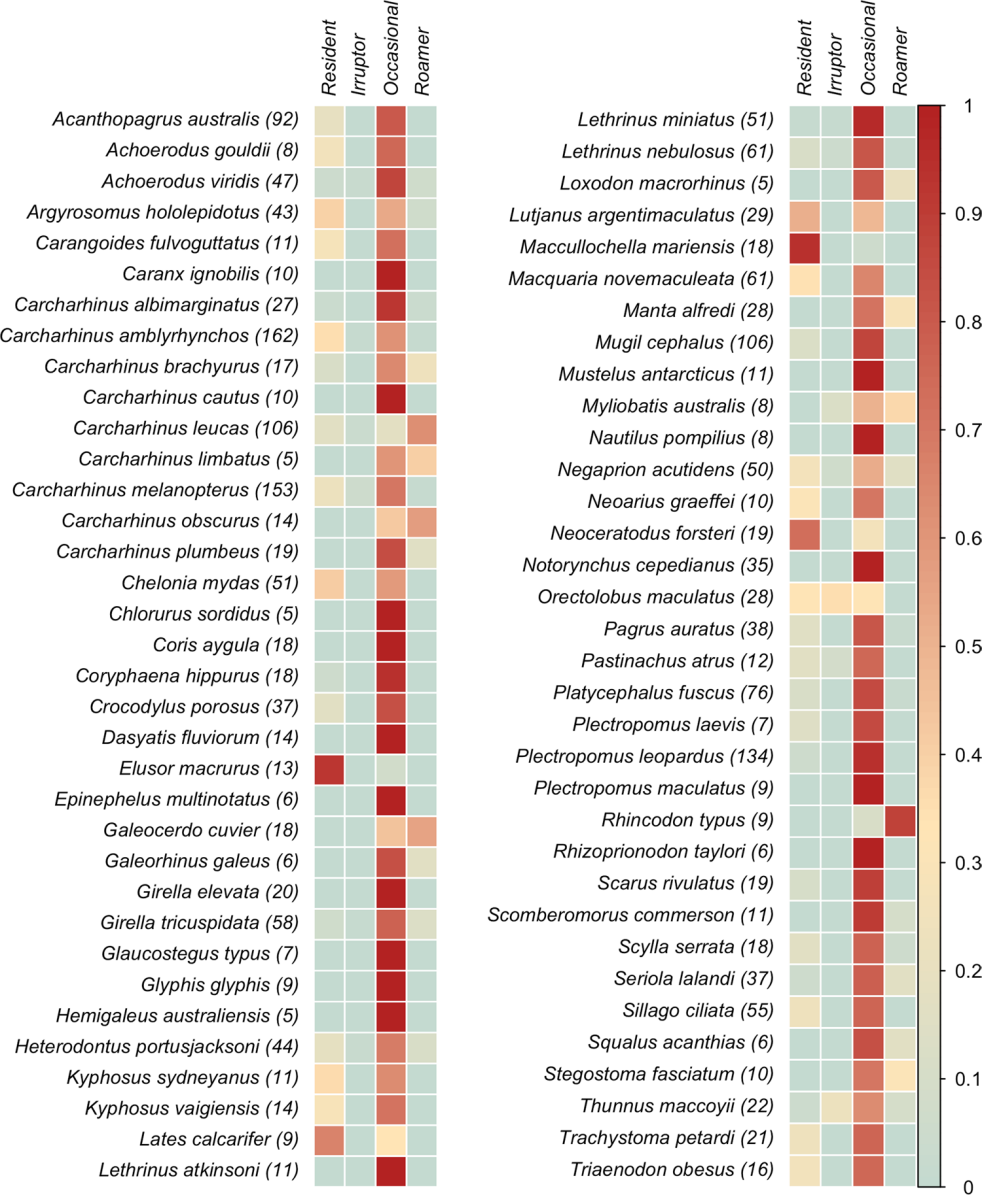
Species in the IMOS ATF database with the color bar indicating the proportion of individuals in each functional movement class (FMC). Parentheses after species name indicate the total number of individuals tagged for that species. Only species with detection data for more than four individuals are displayed.

Cluster analysis of acoustic telemetry detections revealed four distinct clusters, as determined by a gap statistic (Fig. 2a; Table S3). These clusters were considered to represent functional movement classes (FMC) and a posteriori described as ‘Residents’, ‘Occasionals’, ‘Irruptors’, and ‘Roamers’ (Figs 2a and 3; Table S3) rather than described numerically. Residents were detected frequently (mean 68 807 ± SE 4025 detections) on a single installation or a limited number of near (mean 0.8 ± SE 0.04 km) installations, but not further afield, representing site-attached individuals with low levels of dispersal (Fig. 3; Table S3). This FMC is exemplified by mangrove jack (*Lutjanus argentimaculatus*) (Fig. 4). Occasionals were detected infrequently (mean 3441 ± SE 134 detections) and only on a single installation or a limited number of near (mean 2 ± SE 0.1 km) installations (Fig. 3; Table S3). Occasionals comprised site-attached individuals with a medium level of dispersal such as the reef dwelling black drummer (*Girella elevata*) (Fig. 4).

Irruptors were detected frequently (mean 34 176 ± SE 15 439 detections) on a limited number of near installations, but also occasionally on distant (mean 58 ± SE 28 km) installations (Fig. 3; Table S3). Irruptors included site-attached individuals that sometimes undertake long-distance movements, such as spotted wobbegong (*Orectolobus maculatus*; Fig. 4). Roamers were detected across a number of distant (mean 108 ± SE 14 km) installations (Fig. 3; Table S3), and included nomadic individuals continually moving over a large geographical area, such as whale shark (*Rhincodon typus;* Fig. 4).

Residents and Roamers were the most distinguishable FMCs and differentiated from other FMCs by a high number of detections (54% contribution; Table S4) and 99% quantile of distances moved (50% contribution; Table S4), respectively (Figure S1). Occasionals were characterized by the mean time between detections (53% contribution; Table S4) and grouped close to Irruptors, that were characterized by high 99% quantile of distances moved (24% contribution, Table S4) and number of detections (22% contribution, Table S4; Figure S1). The number of clusters was sensitive to the data included, with no stabilization of cluster number when 1, 10, and 100 individuals were randomly removed (Figure S2). The number of individuals and species in each FMC were not equally distributed, with most species (n = 90) and individuals (n = 1578) classified as Occasionals and the least individuals (n = 45) and species (n = 15) classified as Irruptors. Residents contained 393 individuals from 44 species, and Roamers 165 individuals from 29 species.

While many species (36 species; 39%) were exclusively classified within one FMC (e.g. coral trout *Plectropomus leopardus*, red throat emperor *Lethrinus miniatus*), nine species (10%) had individuals in all four FMCs (e.g. grey reef shark *Carcharhinus amblyrhynchos*; Fig. 4). Of the 56 species (61%) with individuals in more than one FMC, most individuals were in one FMC with a few in another (e.g. mullet *Mugil cephalus*, yellowfin bream *Acanthopagrus australis*, Port Jackson shark *Heterodontus portusjacksoni*; Fig. 4). Few species classified homogeneously across multiple FMCs (e.g. spotted wobbegong, mangrove jack; Fig. 4). The proportion of individuals in each FMC should be interpreted with caution where only a few individuals (<5) were tagged (e.g. great hammerhead *Sphyrna mokarran*; Figure S3).

### Effectiveness of the National Network

FMCs were further examined to test whether they persisted across IMOS and non-IMOS installations. Clustering of the Full installation revealed four distinct FMCs, but by contrast a gap statistic did not identify any clusters when either the IMOS, or the non-IMOS installations were individually examined. Furthermore, when IMOS or non-IMOS installations were independently analyzed and required to have four clusters, the clustering became ambiguous with increased overlap between clusters (Fig. 2b,c) compared to the clustering of the Full installation (Fig. 2a).

**Figure 5:**
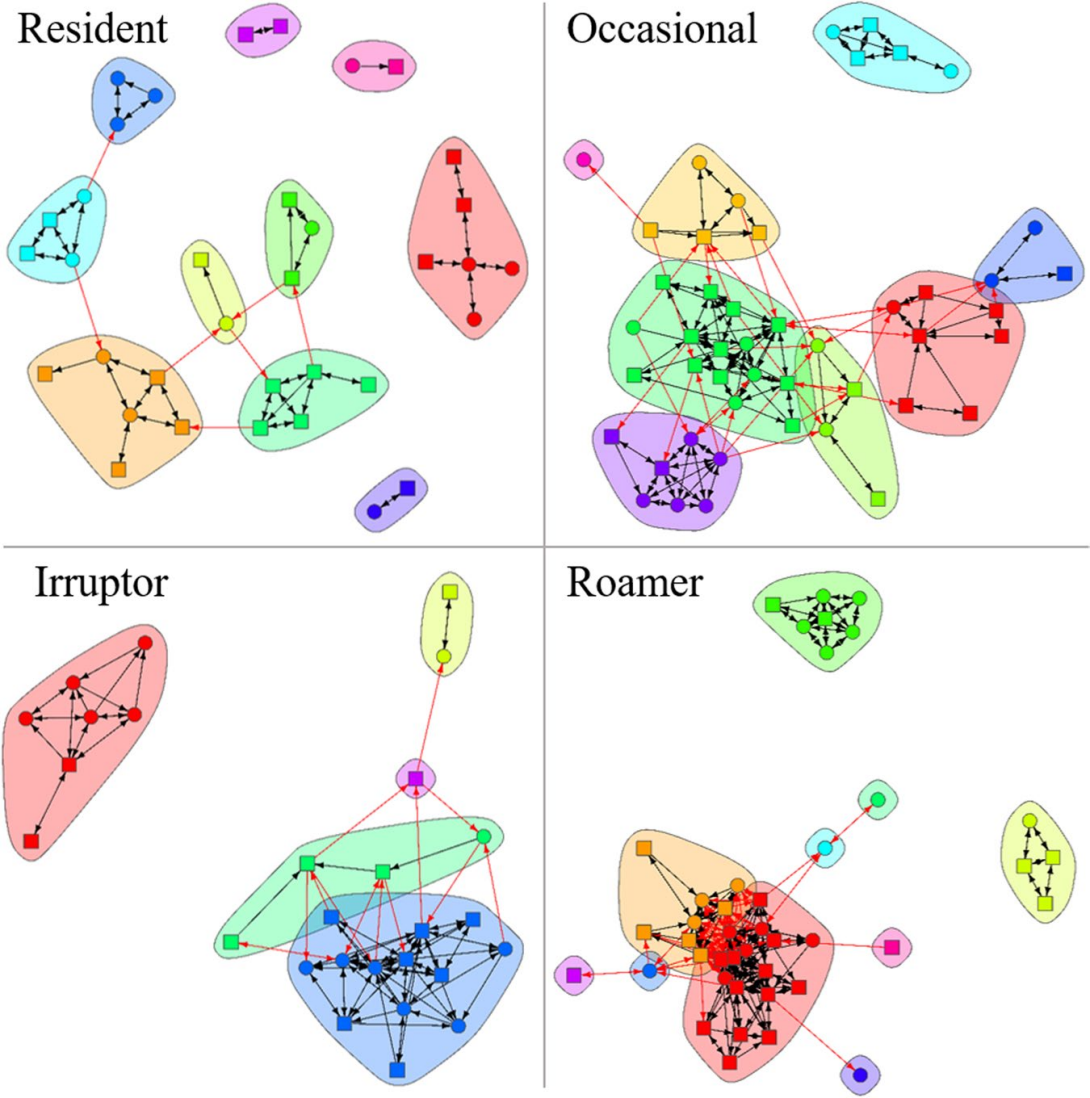
Full installation networks for each functional movement class (FMC), with colors indicating unique clusters of closely related installations. Colors between FMCs are not related, and cluster shapes are arbitrary. Arrows indicate a connection across installations between (red) and within (black) clusters.

Network connectivity of the Full installation type between FMCs was assessed. Each of the FMCs yielded networks with different structural properties that resulted from their different movement types (Fig. 5). Residents yielded a network with a high number of poorly connected installation clusters due to limited movement between installations. Occasionals had a high number of clusters that were highly connected due to medium level dispersal of individuals. Irruptors had a low number of installation clusters because of the low frequency of longer distance movements between installations. Roamers had a high number of partially connected clusters due to large-scale movements between installations.

## Discussion

### Effectiveness of the National Network

The IMOS ATF produces continental-scale data on the movement patterns of marine animals. Our analyses found that the network was more effective in monitoring movement across a diverse range of fauna (92 species) than the individual research installations (non-IMOS) or the national network (IMOS) alone. Only by combining these two differently coordinated and managed installation types into a single facility, connected through a unified database, broad scale ecosystem level research outputs were achieved. The strategic placement and design of IMOS installations along Australia’s coastline were designed to provide more than a random influx of installations, and as a result provide targeted and effective installations that best compliment individual research projects (non-IMOS)^11^. As a result, IMOS ATF is more than just the sum of its parts. Network analyses revealed that IMOS installations perform a critical function in the facility, based on their strategic positioning and high connectivity in the national network, whilst the non-IMOS installations provide finer scale movement information. Thus, both IMOS and non-IMOS installations function to support and enhance one another. Our analyses also demonstrated that removal of either IMOS or non-IMOS installations produce a less well-connected system and reduce the ability of sub-networks to define functional movement classes (FMCs).

The majority of acoustic telemetry studies are designed to examine species-, location-, or system-specific research questions^1^ and are based on current knowledge or researcher expertise relating to the study species. Installations are therefore designed to maximize detection of individuals within the study region. However, the majority of acoustic telemetry publications reveal some individuals are detected for short periods, or not at all, suggesting movement outside the initial study sites. Individuals with few or no detections are typically excluded from analyses in regional-scale studies due to lack of data^15,16^. These individuals may in fact be exhibiting behaviors significant to the dispersal and resilience of the population. We found that many species fell within more than one FMC, demonstrating that the IMOS ATF detected movements of a few tagged individuals outside the movements of the general population, enhancing research by revealing the existence of occasional long-range movements. The ability to capture these rare movements helps explain the fate of some low detection individuals and reduces uncertainty in movement and dispersal predictions for a species.

An extensive network provides the capacity to understand movements beyond previous expectations and assumptions of researchers when developing single-species, single-location studies. The positioning of acoustic receivers does play an important role in the efficacy of the national network, as demonstrated here. This is primarily due to the imperfect nature of data collection through passive acoustic networks, with the probability of tag detection related to the presence of an acoustic receiver. There are recommended approaches to deploying receiver arrays, and many non-IMOS arrays follow these recommendations which ultimately helps to standardize data collection^17^. Network analysis results highlight installation connectivity across geographic regions and between FMCs to reveal linkages between installations despite variation in array design and receiver location. These results can be used to expand or improve array placements for future animal movement monitoring.

### Management Implications of Functional Movement Class Identification

The identified FMCs revealed various spatial patterns for species ranging from localized to continental-scale movements. A species with a high degree of fidelity to an area is expected to play a very different role in an ecosystem than a highly mobile nomadic species that may move through that area only occasionally^4^. Managing or conserving such different species is likely to require different approaches and would need to be based upon an understanding of the areas occupied and the level of connectivity throughout the extent of their geographical range. Management objectives and actions often rely on the predictability of species presence, abundance, and movement^3^. While this is generally true, there can be substantial intra-specific individual variation, as seen here. The scale of the IMOS ATF network provides the capacity to determine the extent and scale of dispersal movements and habitats used. For example, large-scale arrays are capable of measuring inter-specific variation in habitat use, the results of which can be used to inform the design of marine protected areas^15,18,19^. As a result, large-scale networks can improve our understanding of the ecological role and management needs of a population or species.

Determining FMCs using metrics based on movements and detectability of acoustic transmitters successfully identified four distinct and ecologically valuable classes across 92 species. However, sensitivity analysis showed that the number of clusters was sensitive to the data included in analysis. This is likely related to the sensitivity of the *k*-means algorithm to noise and outliers^20,21^. Acoustic telemetry data is often noisy due to the low probability of detecting a tagged individual^17^. While clustering results were sensitive to the data included, we must acknowledge that clustering analyses are exploratory tools and must be adapted to application specific use^20^. In this sense, *k*-means clustering was highly suitable to our study as partitioning methods handle large data sets better than hierarchical clustering, and are also unaffected by data order^20,21^. For these reasons, our approach to classifying animal movement is likely applicable to other acoustic telemetry datasets due to the similar nature of data collection. As such, there is great potential for future comparative studies across local to global scales, however future work may not find data segregates into four clusters, especially if the spatial scale of data is limited. We recommend analyses are conducted and interpreted on a case-by-case basis, rather than matched a priori to the results seen here. Furthermore, the classification of movement behavior is also dependent on data collection methods, where other biologging approaches could sample different behavior (e.g. Abrahms, *et al*.)^4^. Ultimately, the aim of cluster methods is to find structure in the data, and our approach was successful in identifying inter-specific movements and residency of marine animals.

The potential utility of using behavioral information such as FMCs to inform conservation planning and management of aquatic animals is high. For example, Green, *et al*.^22^ distinguished three movement types of fish in order to provide advice on marine spatial planning. Here, we found that some FMCs encompass all individuals of a given species and thus reveal their vulnerability. For instance, highly resident animals (e.g. mangrove jack) are likely to be impacted by both localized disruptions and longer-term environmental change due to their restricted movements. In contrast, FMCs also revealed hitherto unforeseen plasticity in some populations and species. For instance, both great hammerhead and spotted wobbegong were found in three FMCs; Residents, Occasionals, and Irruptors, and these divergent movement strategies may provide a buffer for at least some of the population in a changing environment. Measuring such intra-specific variability in movement patterns can inform the spatiotemporal scale and type of management response. For example, individuals with high site fidelity may be best protected using static spatial zoning such as marine protected areas. Whereas individuals with low site fidelity and the capacity to make large migrations could be protected through traditional fisheries management techniques (i.e. bag, size, and quota limits), or through novel strategies such as dynamic ocean management^23^. Species with high variability in movement may require a suite of management approaches including cross-jurisdictional coordination if a significant number of individuals show Irruptor or other broad movement patterns. Inclusion of acoustic FMC data may play a key role in enhancing management approaches.

## Conclusion

The use of a continental-scale collaborative monitoring system revealed emergent properties of individual-based movement of marine fauna and therefore improved potential to address contemporary research and conservation issues. Hays, *et al*.^24^ identified 10 key questions to advance the study of movement ecology of marine megafauna research. The approach outlined here allows three of the most important of these to be addressed on a continental-scale: How will climate change impact animal movements? How do anthropogenic activities (e.g. shipping, fishing, and water management) affect movements? What areas can be considered hotspots for multiple species on a global scale? The applicability of the IMOS ATF network to these globally relevant research questions highlights the utility and capacity of this approach to advance research, management, and conservation efforts within and beyond Australia.

## Methods

### Acoustic Telemetry Detection Data

Acoustic telemetry data for analysis was extracted from the IMOS ATF data repository, and further processed for quality control (QC flags 1 and 2)^12^. Tag detection data contained a timestamp, coordinate location of the receiver, and additional metadata (e.g. species name). Receiver stations were identified as being IMOS or non-IMOS installations (i.e. independent research project). This classification resulted in three installation types; ‘IMOS’, ‘non-IMOS’, and ‘Full’ (both IMOS and non-IMOS installations).

### Network Analyses

Network analyses were used to assess the utility and redundancy of the three installation types in the IMOS ATF (‘Full’, ‘IMOS’, ‘non-IMOS’), where an installation was considered to be a group of receivers deployed in a specific region. Detection data were used to create square matrices that counted relative movements between installations, with relative movement defined as the number of times individuals moved between two installations divided by the total number of movements made by the individuals^25^. Three relative movement networks were created according to the installation types described above. Each relative movement network was assessed at the network and installation level using 14 metrics (supplementary material). The relative movement networks and metrics were examined to determine whether IMOS installations were central to each constructed network and if their removal decreased connectivity between all installations. Analyses were conducted using the *igraph*^26^ and *sna*^27^ packages in R^28^ (supplementary material).

### Functional Movement Classes

A cluster analysis was used to classify animal movement patterns across the Full continental-scale facility (i.e. including IMOS and non-IMOS installations). The cluster analysis used seven covariates to classify animal movements: the number of installations at which a transmitter was detected, number of detections, mean time between detections (min), and four quantiles (25%, 50%, 75%, 99%) of distance travelled (km) between consecutive detections. A quantile of 99% was considered a better metric of the distribution of movement data, instead of a 100% quantile that would only indicate the maximum distance moved. All covariates were square-root transformed to reduce skewness in the data, centered, and scaled to achieve homoscedasticity prior to analysis. Clustering was done using *k*-means clustering in R, with the optimum number of clusters determined using the gap statistic – a goodness of clustering measure^29^. The optimal number of clusters were further examined a posteriori to define functional movement classes (FMC). Clusters were visualized with Principle Components Analysis (PCA) using the *factoextra* package^30^ in R. The sensitivity of the gap statistic to the data included was examined by randomly excluding 1, 10, and 100 tags, with the mean number of clusters determined after 20 iterations. The covariates driving the difference between FMCs were identified using similarity percentages (SIMPER) analysis based on a Euclidean distance measure in PRIMER v.7^31^. Data were transformed using standard dispersion weighting and log(*x* + 1) determined by shade plots^32^, and visualized using PCA Ordination.

### Effectiveness of the National Network

FMCs were further examined to test whether they persisted across IMOS and non-IMOS installations, and to assess network connectivity of the Full installation between FMCs. Three approaches were taken: (1) the gap statistic was re-evaluated for detection data from either IMOS or non-IMOS installations alone to determine the optimal number of functional movement classes for each installation type; (2) *k*-means clustering was repeated using detection data from either IMOS or non-IMOS installations, but using the same number of clusters as indicated by the Full installation; (3) a network analysis using the methods outlined above was conducted for each FMC to assess how connectivity across the Full installation differed between FMCs.

### Data Availability

The datasets analyzed during the current study are available from the acoustic tracking database (https://animaltracking.aodn.org.au) of the Integrated Marine Observing System Animal Tracking Facility (IMOS ATF; www.imos.org.au).

## Electronic supplementary material

Supplementary Information
