# Supplementary material for: Continental-scale animal tracking reveals functional movement classes across marine taxa
Source: Sci Rep. 2018 Feb 27;8:3717. doi: 10.1038/s41598-018-21988-5 (PMC5829234; doi:10.1038/s41598-018-21988-5)
Supplement: Supplementary file 1 — Supplementary Information [file 41598_2018_21988_MOESM1_ESM.doc]

***Supplementary Information***

**Continental-scale animal tracking reveals functional movement classes across marine taxa**

Stephanie Brodie*, Elodie J. I. Lédée, Michelle R. Heupel, Russell C. Babcock, Hamish A. Campbell, Daniel C. Gledhill, Xavier Hoenner, Charlie Huveneers, Fabrice R. A. Jaine, Colin A. Simpfendorfer, Matthew D. Taylor, Vinay Udyawer, Robert G. Harcourt

* Corresponding Author: stephanie.brodie@unsw.edu.au

**Supplementary Methods**

*Description of network analysis*

Each relative movement network was assessed at the network and installation level using 14 metrics (i.e. 8 network and 6 centrality metrics). Analyses were conducted using the igraph R package (Csárdi & Nepusz 2006) and sna R package (Butts 2013). Network visualisations were made using the igraph R package (graph.empty, vertices, edges, and plot functions). The igraph R package functions were used to calculate node strength, eigenvector, closeness, density, diameter, average path length, modularity and communities. While the sna R package functions were used to calculate in- and out-degree, betweenness, and closeness.

Network metrics: Eight metrics including number of installations, paths, average path length, community (number and modularity), subgraph, density, and diameter. A path was a route between two installations in the network. Average path length (APL) was a measure of separation within the network (Rayfield et al. 2011). Community classified densely connected installations into sub-groups within the network via random walks (i.e. movement between two subgroups was possible - Csárdi & Nepusz 2006, Bodin et al. 2011). Community measured number of communities within and modularity score of the partitioning for each network. Subgraph identified the number of sub-networks and/or isolates (installation not connected to any other) that are disconnected from the rest of the network (i.e. movement between two components was not possible) and represented the level of network fragmentation (Bodin et al. 2011). Density measured the proportion of edge (or route) present in the network out of a total number of possible edge (ranging from 0 to 1, with a value of 1 when all arrays in the network are connected to all others). Lastly, diameter indicated the network size as the longest path between any pair of arrays in the network (Urban & Keitt 2001).

Centrality metrics: Six centrality metrics (betweenness, closeness, eigenvector, node strength, in- and out-degree) were calculated for each installation within each network. Betweenness indicated installation connectivity (i.e. how much an installation was involved in the flow of individuals across or around the area of interest (Minor & Urban 2007). Closeness measured how central an installation's position was in network space (i.e. smallest number of paths linking installations, i.e. geodesic distance - Urban et al. 2009). Eigenvector indicated how strategically placed an installation was within the network (Bodin et al. 2011). Lastly, node strength was a measure of the connection weight at each installation (Barrat et al. 2004) and in-degree and out-degree were a measure of incoming and out-going connection, respectively.

**Supplementary Figures and Tables**

**Table S1** Centrality metrics for 48 installations analyzed using network analysis, indicating In degree (ID), Out degree (OD), Node strength (NS), Eigenvalue (E), Betweenness (B), and Closeness (C). Data sorted by Eigenvalue. Installation names in italics indicate IMOS only installations.

| **Installation** | **ID** | **OD** | **NS** | **E** | **B** | **C** |
| --- | --- | --- | --- | --- | --- | --- |
| Ningaloo Shark | 0.3623 | 0.3623 | 6 | 0.7102 | 50.0000 | 0.0088 |
| CSIRO Mangrove Bay | 0.3658 | 0.3658 | 13 | 0.7040 | 109.5000 | 0.0085 |
| *ATF Ningaloo* | 0.0026 | 0.0027 | 14 | 0.0050 | 119.9144 | 0.0100 |
| *ATF Ningaloo North line* | 0.0008 | 0.0008 | 13 | 0.0013 | 59.7939 | 0.0093 |
| *ATF Coral Bay* | 0.0201 | 0.0202 | 15 | 0.0007 | 16.4167 | 0.0097 |
| *ATF Ningaloo Central line* | 0.0003 | 0.0003 | 11 | 0.0002 | 49.0000 | 0.0074 |
| *ATF Ningaloo South line* | 0.0196 | 0.0196 | 10 | 5.20E-05 | 218.2083 | 0.0073 |
| NSW DPI Port Stephens | 0.0002 | 0.0002 | 28 | 6.51E-06 | 257.0258 | 0.0115 |
| NSW DPI Sydney | 0.0045 | 0.0046 | 35 | 6.46E-06 | 105.9563 | 0.0128 |
| *ATF Sydney* | 0.0048 | 0.0048 | 32 | 3.76E-08 | 8.3141 | 0.0119 |
| NSW DPI Clyde | 0.0039 | 0.0039 | 12 | 2.36E-08 | 19.3702 | 0.0095 |
| *ATF Cabbage* | 0.0076 | 0.0077 | 37 | 2.10E-08 | 183.4702 | 0.0127 |
| *ATF Bondi line* | 0.0012 | 0.0011 | 36 | 8.90E-09 | 468.7031 | 0.0132 |
| NSW DPI Coastal | 0.0022 | 0.0023 | 41 | 7.49E-09 | 147.9860 | 0.0130 |
| *ATF Rowley* | 6.64E-06 | 1.99E-05 | 5 | 6.60E-09 | 11.3333 | 0.0099 |
| NSW DPI Gates | 0.0008 | 0.0008 | 38 | 3.41E-09 | 217.8745 | 0.0127 |
| NSW DPI Clarence | 0.0027 | 0.0027 | 14 | 2.97E-09 | 14.7262 | 0.0095 |
| MQ Bronte-Coogee | 0.0095 | 0.0095 | 31 | 2.52E-09 | 184.3583 | 0.0111 |
| NSW DPI Artificial | 0.0021 | 0.0021 | 19 | 2.08E-09 | 8.2000 | 0.0108 |
| *ATF Port Stephens* | 0.0001 | 0.0001 | 13 | 1.18E-09 | 9.3333 | 0.0102 |
| NSW DPI Shoalhaven | 0.0021 | 0.0022 | 17 | 6.13E-10 | 40.3409 | 0.0099 |
| CSIRO: Animal | 8.64E-05 | 6.98E-05 | 21 | 2.97E-10 | 81.5384 | 0.0111 |
| NSW DPI Drummer | 6.31E-05 | 5.65E-05 | 15 | 1.81E-10 | 54.8167 | 0.0097 |
| Seven Gill tracking | 0 | 1.99E-05 | 4 | 1.78E-10 | 0 | 0.0082 |
| Townsville Reefs | 0.0002 | 0.0002 | 29 | 1.20E-10 | 230.4098 | 0.0122 |
| *ATF One Tree* | 0.0016 | 0.0016 | 20 | 1.19E-10 | 154.9151 | 0.0114 |
| NSW DPI Jervis Bay | 0.0001 | 0.0002 | 19 | 6.10E-11 | 136.0914 | 0.0102 |
| JCU Moreton Bay | 0.0001 | 8.64E-05 | 24 | 6.07E-11 | 188.8400 | 0.0112 |
| *ATF Narooma* | 0.0002 | 0.0001 | 17 | 6.03E-11 | 46.6549 | 0.0098 |
| *ATF Coffs* | 0.0003 | 0.0002 | 25 | 7.12E-12 | 13.2152 | 0.0115 |
| *ATF Heron* | 0.0039 | 0.0038 | 33 | 2.63E-12 | 652.3899 | 0.0123 |
| Wenlock River | 0.0001 | 0.0001 | 5 | 7.18E-13 | 0 | 0.0090 |
| UQ Project Manta | 5.65E-05 | 6.64E-05 | 22 | 2.77E-13 | 180.9138 | 0.0114 |
| *ATF Orpheus* | 0.0881 | 0.0880 | 13 | 8.41E-14 | 239.6519 | 0.0106 |
| *ATF Maria Island* | 1.33E-05 | 6.64E-06 | 6 | 8.20E-14 | 52.6721 | 0.0088 |
| UQ grey nurse | 4.65E-05 | 3.32E-05 | 9 | 3.23E-14 | 21.3406 | 0.0100 |
| JCU Pioneer Bay | 0.0880 | 0.0880 | 2 | 2.06E-14 | 0 | 0.0071 |
| Spangled Emperor | 0.0022 | 0.0022 | 3 | 1.94E-14 | 0 | 0.0082 |
| *AIMS Scientific* | 1.66E-05 | 1.66E-05 | 7 | 4.40E-15 | 27.1774 | 0.0099 |
| *ATF Cape Barron Island* | 1.33E-05 | 9.97E-06 | 6 | 1.11E-15 | 24.2632 | 0.0072 |
| Logan River | 0.0005 | 0.0005 | 4 | 5.53E-16 | 46.2576 | 0.0083 |
| Logan River TERN | 0.0005 | 0.0005 | 3 | 5.53E-16 | 0 | 0.0075 |
| CSIRO Gladstone | 3.32E-06 | 6.64E-06 | 3 | 5.52E-16 | 4.5409 | 0.0081 |
| Gulf St Vincent NE | 0.0002 | 0.0001 | 10 | 2.42E-17 | 393.0000 | 0.0084 |
| Gulf St Vincent Metro | 0.0001 | 0.0001 | 6 | 5.52E-21 | 0 | 0.0061 |
| Gulf St Vincent NW | 7.97E-05 | 8.64E-05 | 6 | 4.64E-21 | 138.0000 | 0.0061 |
| *ATF Glenelg* | 6.31E-05 | 6.64E-05 | 6 | 4.43E-22 | 225.0000 | 0.0061 |
| *ATF Perth line* | 3.32E-06 | 3.32E-06 | 2 | 2.21E-22 | 0 | 0.0061 |

**Table S2** Network metrics for the three installation type networks.

|  | **Installation Type** | | |
| --- | --- | --- | --- |
|  | **Full** | **IMOS** | **non-IMOS** |
| Number of Installations | 48 | 18 | 26 |
| Paths | 380 | 73 | 132 |
| Diameter | 5 | 4 | 26 |
| Average Path Length | 3.432 | 3.15 | 10.723 |
| Density | 0.168 | 0.239 | 0.203 |
| Subgraph | 1 | 1 | 2 |
| Communities | 6 | 4 | 5 |
| Modularity | 0.238 | 0.365 | 0.185 |

| **FMC** | **Description** | **99%** | **D** | **I** | **∆t** | **Example species** |
| --- | --- | --- | --- | --- | --- | --- |
| Resident | Site-attached individuals with low levels of dispersal. | 0.8 ± 0.04 | 68 807 ± 4025 | 1.5 ± 0.04 | 8 ± 0.2 | Mangrove jack (*Lutjanus argentimaculatus*) |
| Occasional | Site-attached individuals with a medium level of dispersal. | 2 ± 0.1 | 3441 ± 134 | 1.4 ± 0.02 | 11 ± 0.1 | Black drummer  (*Girella elevate*) |
| Irruptor | Site-attached individuals that sometimes undertake long-distance movements. | 58 ± 28 | 34 176 ± 15 439 | 2.9 ± 0.6 | 17 ± 2 | Spotted wobbegong (*Orectolobus maculatus*) |
| Roamer | Nomadic individuals continually moving over a large geographical area. | 108 ± 14 | 2678 ± 422 | 7.2 ± 0.4 | 13 ± 0.6 | Whale shark  (*Rhincodon typus*) |

**Table S3** Summary of functional movement classes (FMC) including FMC name and description, and covariate mean ± SE across individuals: 99% movement quantile (99%; km), number of detections (D), number of installations (I), and mean time between detections (∆t; min).

**Table S4** Percentage contribution of each covariate characterizing the functional movement classes.

| **Covariates** | **Irruptor** | **Occasional** | **Roamer** | **Resident** |
| --- | --- | --- | --- | --- |
| 25% quantiles | 6.19 | 0 | 0 | 0 |
| 50% quantiles | 2.60 | 0.06 | 0.04 | 0.13 |
| 75% quantiles | 17.30 | 8.84 | 9.87 | 1.55 |
| 99% quantiles | 23.83 | 2.20 | 49.79 | 0.08 |
| Time difference | 10.76 | 52.91 | 12.31 | 30.29 |
| Number of installations | 17.27 | 27.44 | 26.68 | 13.70 |
| Number of detections | 22.05 | 8.55 | 1.30 | 54.25 |


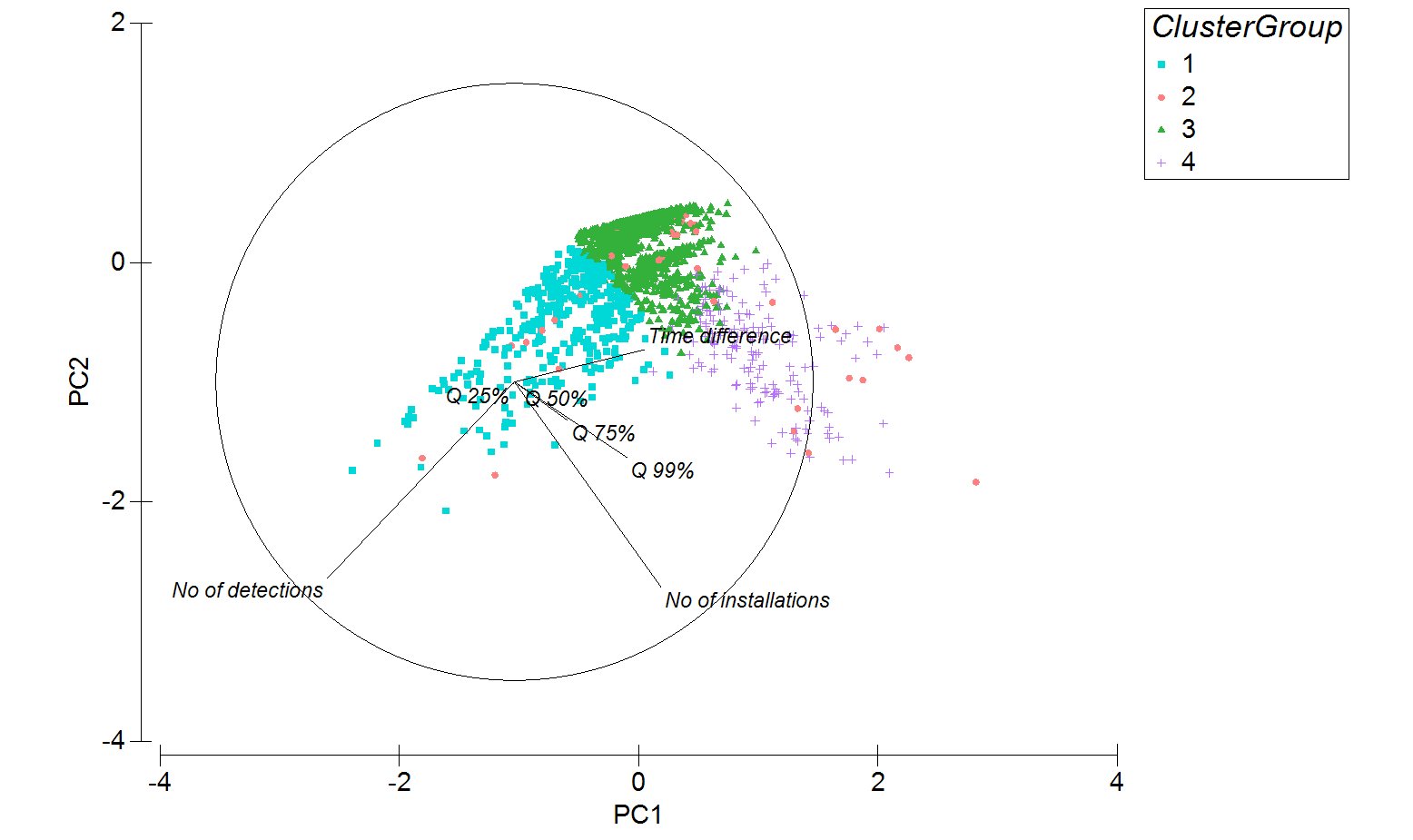


**Figure S1** Principal Component Analysis Ordination of Functional Movement Classes (FMC), with vectors indicating strength and direction of Pearson correlations. Red circles are Irruptors, green triangles are Occasionals, blue squares are Residents, and purple crosses are Roamers.


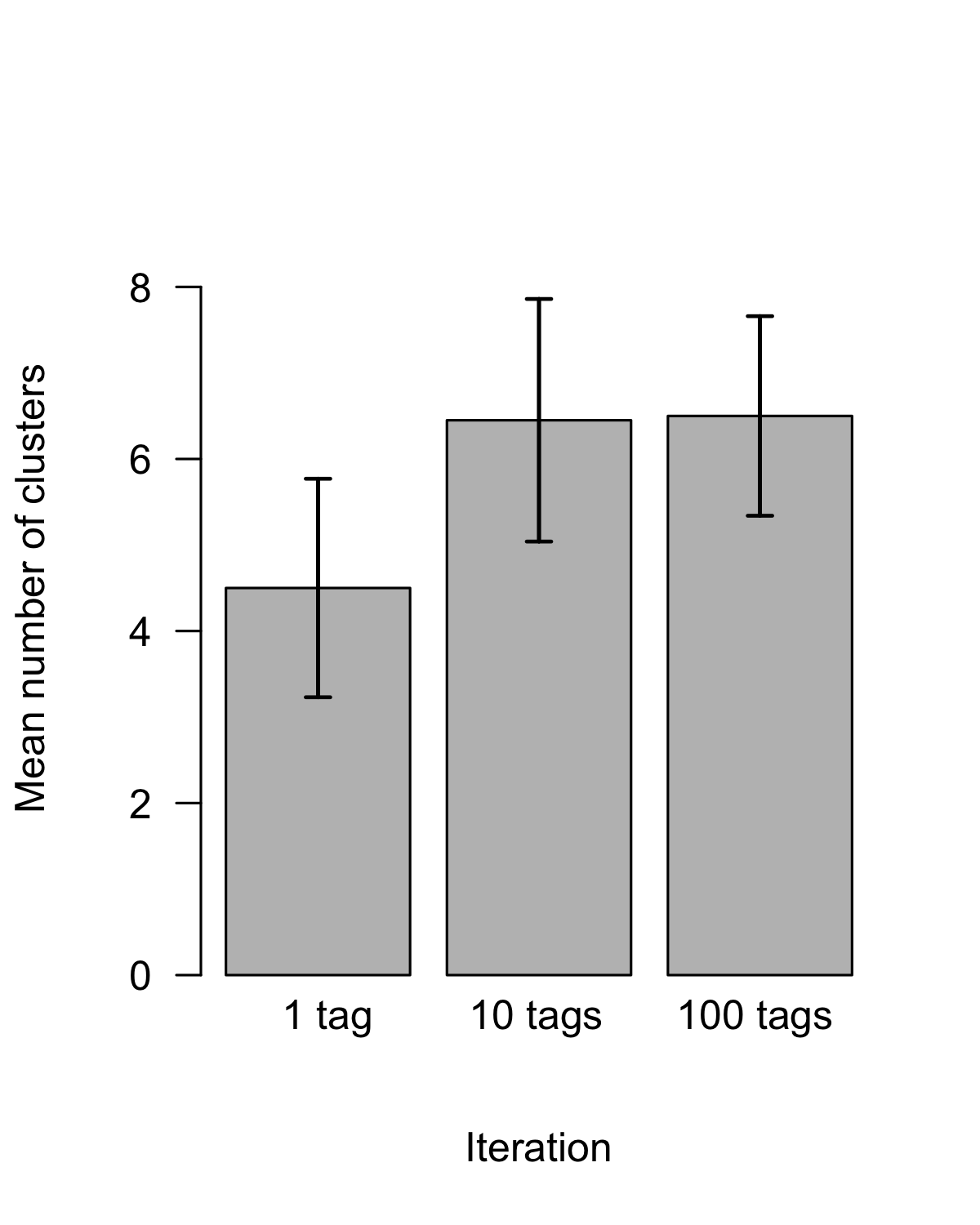


**Figure S2** Mean (±S.E.) number of clusters when 1, 10, or 100 individuals were randomly removed from analysis. Simulations were conducted 20 times and clusters were determined from the gap statistic.


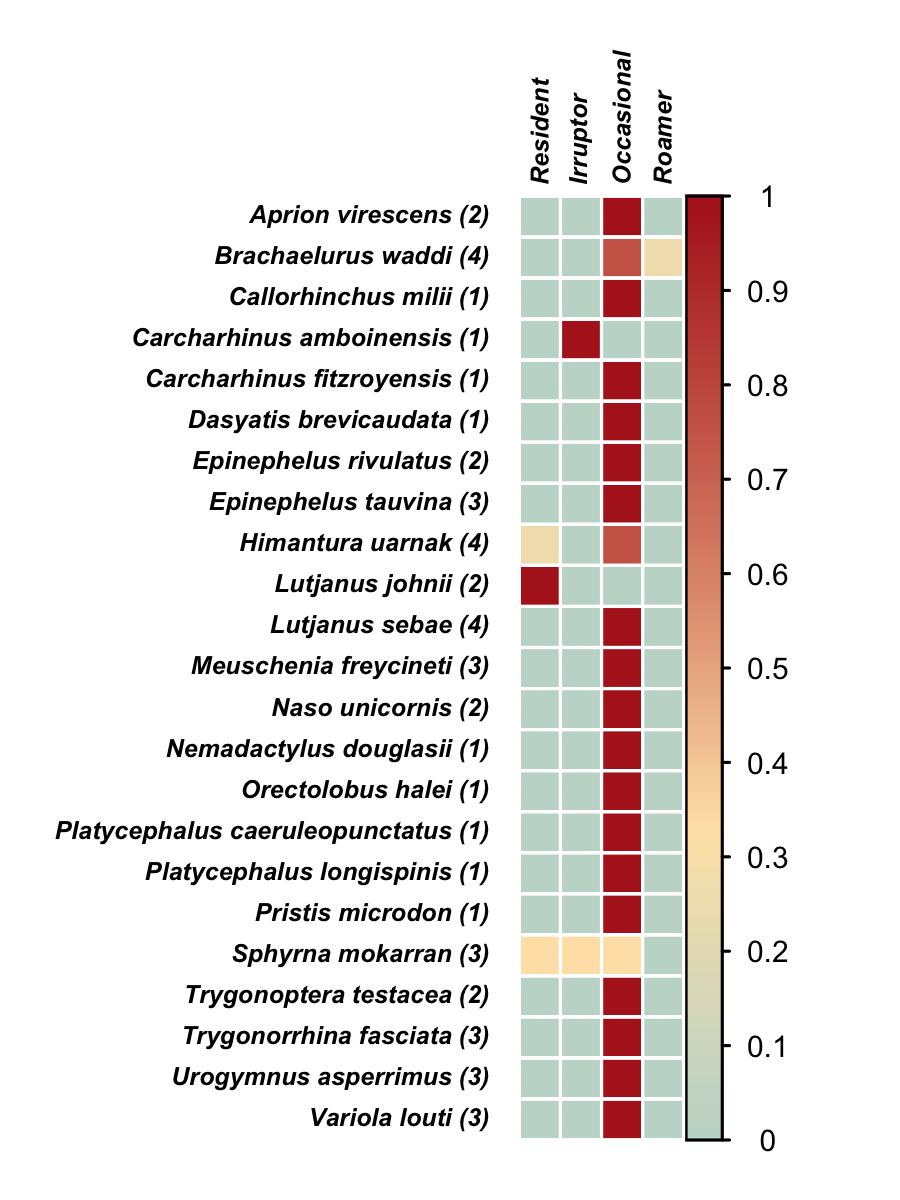


**Figure S3** Species in the IMOS ATF database with less than five individuals. Color bar indicates the proportion of individuals in each Functional Movement Class (FMC). Parentheses after species name indicate the total number of individuals tagged for that species.

**Supplementary References**

Barrat, A., M. Barthelemy, R. Pastor-Satorras and A. Vespignani (2004). "The architecture of complex weighted networks." Proceedings of the National Academy of Sciences of the United States of America, 101(11): 3747-3752.

Bodin, Ö., S. Ramirez-Sanchez, H. Ernstson and C. Prell (2011). Some basic structural characteristics of networks. Social Networks and Natural Resource Management - Uncovering the Social Fabric of Environmental Governance.(Ö. Bodin and C. Prell).29-43 United Kingdom at the University Press, Cambridge, Cambridge University Press.

Butts, C. T. (2013). “sna: Tools for Social Network Analysis.” R package version 2.3-1. http://cran.r-project.org/package=sna

Csárdi, G. and T. Nepusz (2006). "The igraph software package for complex network." InterJournal Complex Systems: 1695.

Minor, E. S. and D. L. Urban (2007). "Graph theory as a proxy for spatially explicit population models in conservation planning." Ecological Applications 17(6): 1771-1782.

Rayfield, B., M. J. Fortin and A. Fall (2011). "Connectivity for conservation: a framework to classify network measures." Ecology 92(4): 847-858.

Urban, D. L. and T. Keitt (2001). "Landscape connectivity: A graph-theoretic perspective." Ecology 82(5): 1205-1218.

Urban, D. L., E. S. Minor, E. A. Treml and R. S. Schick (2009). "Graph models of habitat mosaics." Ecology Letters 12(3): 260-273.
